# The synergy of transcriptional and epigenetic controls promotes fruit ripening in peach

**DOI:** 10.1093/plphys/kiad663

**Published:** 2023-12-14

**Authors:** Yee-Shan Ku

**Affiliations:** Assistant Features Editor, Plant Physiology, American Society of Plant Biologists; School of Life Sciences and Centre for Soybean Research of the State Key Laboratory of Agrobiotechnology, The Chinese University of Hong Kong, Hong Kong SAR, China

Fruit ripening is a complex process involving hormonal, transcriptional, and epigenetic regulations. During ripening in many fruits, 1-aminocyclopropane-1-carboxylic acid is converted to ethylene, which binds to ethylene receptors to activate genes such as those encoding ERF (ethylene response factor) transcription factors (TFs) (Liu et al. 2020). ERF TFs then regulate the expressions of fruit ripening–related genes such as those regulating fruit softness, color, and flavor (Gao et al. 2020). Early studies on fruit ripening predominantly employed tomato as the model. Later, the fruitENCODE project annotated genes and revealed the epigenomes of other climacteric fruits, including peach (Lü et al. 2018). In peach, NAC TFs were suggested to regulate genes for ethylene biosynthesis (Lü et al. 2018). However, the detailed role of NACs in peach fruit ripening remained largely unknown.

In this issue of *Plant Physiology*, Cao et al. reported the synergistic regulation of peach fruit quality and ripening by the TF PpNAC1 and DNA demethylase PpDML1 (Cao et al. 2023). The expression of *PpNAC1* increases during fruit ripening and remains high after harvest (Cao et al. 2023). Moreover, the expression of *PpNAC1* can be induced by ethylene treatment (Cao et al. 2023). By DNA affinity purification-seq, more than 2,000 candidate target genes were found to be bound at the promoter by PcNAC1 (Cao et al. 2023). The candidate target genes were narrowed down to focus on genes that are positively correlated with ripening-related characters such as ethylene production and sucrose content (Cao et al. 2023).

Gene methylation has been suggested as a contributor to the regulation of fruit ripening (Zhong et al. 2013; Lü et al. 2018). The promoters of fruit ripening–related genes can be methylated at TF binding sites (Zhong et al. 2013). Moreover, the methylation statuses of the TF binding sites are associated with the accurate timing of fruit ripening (Zhong et al. 2013). In this study by Cao et al. (Cao et al. 2023), the authors investigated the methylation status of the target gene promoters. Differentially methylated regions were found at or close to PpNAC1 binding sites, suggesting that DNA methylation may influence the binding of PpNAC1 to the target genes and thereby affect their expression.

To investigate the association between *PpNAC1* and genes regulating genome methylation, the authors overexpressed *PpNAC1* in peach callus and showed an elevated expression of *PpDML1* correlated with the binding of PpNAC1 to the promoter of *PpDML1*, which encodes a DNA demethylase. Further analysis showed that expression levels of ripening-related genes and *PpNAC1* were positively correlated with the expression level of *PpDML1*, and their methylation levels were negatively correlated with *PpDML1* expression. Based on these results, the authors suggested a synergetic fruit ripening regulation brough forth by PpNAC1 and PpDML1. During fruit ripening, the increasing expression of *PpNAC1* promotes the expressions of fruit ripening–related genes, ethylene biosynthesis genes, and *PpDML1*. The increased expression of *PpDML1* leads to further increased expression of *PpNAC1* and its target genes in a positive feedback loop (Figure).

**Figure.**
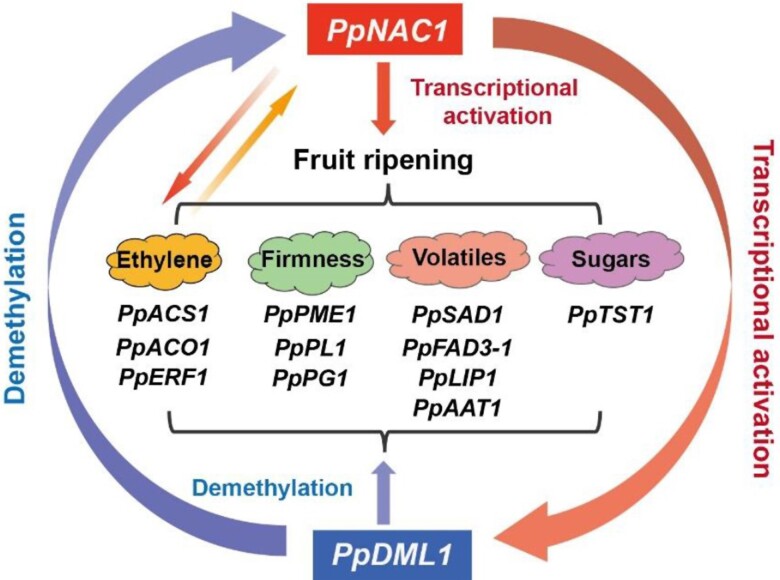
During fruit ripening, PpNAC1 activates the expressions of fruit ripening–related genes, including those related to ethylene biosynthesis, fruit firmness, volatile production, and sugar storage. PpNAC1 also activates the expression of *PpDML1*. The promoted ethylene level and *PpDML1* expression further promote the expression of *PpNAC1*. Moreover, PpDML1 is positively correlated with the demethylation of the promoters of *PpNAC1* and its target genes. The transcriptional activation by PpNAC1 on its target genes is then enhanced by both its elevated expression and the demethylation of the target gene promoters. This figure is adapted from Cao et al. 2023.

In this study, the authors reported the important role of PpNAC1 to regulate multiple fruit ripening–related genes, including those involved in ethylene biosynthesis, fruit softening, volatile synthesis, and sugar storage. The study also showed the synergy of transcriptional and epigenetic controls by the interplay between PpNAC1 and PpDML1. Although tomato has been the model for fruit ripening studies, this study revealed the positive feedback loop that underpins the fruit ripening mechanism in the agroeconomically valuable peach fruit.

## Dive Curated Terms

The following phenotypic, genotypic, and functional terms are of significance to the work described in this paper:

NAC CHEBI: CHEBI:7421

fruit AmiGo: PO:0009001
